# Evaluation of the Physical Health Monitoring Clinic in CAMHS Intellectual Disability Service

**DOI:** 10.1192/j.eurpsy.2026.10433

**Published:** 2026-07-31

**Authors:** B. Kowalewska, S. Sharifuddin, R. S. A. Sae Ali, N. OKeeffe, L. Sharkey

**Affiliations:** 1 CAMHS-ID, HSE, Dublin, Ireland; 2 Psychiatry, Nicolaus Copernicus University, Bydgoszcz, Poland

## Abstract

**Introduction:**

Children and adolescents with intellectual disabilities are at increased risk for a wide range of physical health conditions, including higher rates of obesity, cardiovascular disease, epilepsy, as well as greater vulnerability to adverse effects of antipsychotic medication compared with their typically developing peers. Psychotropic medications, frequently prescribed in this population to manage comorbid psychiatric disorders, carry significant metabolic risks such as weight gain, diabetes, and cardiovascular complications. Regular and systematic physical health monitoring, including metabolic screening, is therefore essential to minimise risks and optimise health outcomes. National guidelines (NICE, STOMP/STAMP) emphasise the importance of regular monitoring and integrated care for this population.

In line with these recommendations, the CAMHS Intellectual Disability service introduced a Physical Health Monitoring Clinic in December 2024. The initiative aims to address gaps in care by providing structured, focusing on early identification and management of physical health concerns and medication-related side effects.

**Objectives:**

The study aimed to evaluate the effectiveness, feasibility, and acceptability of the clinic within the CAMHS-ID service. Objectives included identifying physical health issues, documenting subsequent clinical actions, and exploring caregiver and service user satisfaction. Staff views on clinic operations and barriers were also gathered.

**Methods:**

A mixed-methods approach was adopted. Retrospective and prospective data were collated, with quantitative measures describing attendance, completion of screening protocols, and clinical outcomes. Qualitative data, obtained through caregiver and staff feedback forms, underwent thematic analysis to identify recurrent strengths and challenges.

**Results:**

98% or referred patients attended appointments. Missed appointment were reschedules. Monitoring quality was high: weight, height, and BMI were recorded in 100% of appointments; BP and HR in 95%; and monitoring outcomes were documented in 90% of clinic notes. Electrocardiograms and blood tests were arranged externally through GPs or paediatric services. Feedback indicated high levels of satisfaction among parents, who highlighted accessibility, clarity of communication, and perceived benefit for their child. Some dissatisfaction was noted with clinical room settings and equipment. Staff feedback echoed these findings, emphasising strengths in care coordination, timeliness of assessments, and improved health education for families.

**Conclusions:**

This evaluation demonstrates the value of structured, proactive health screening in CAMHS ID services. The clinic facilitated early detection, supported multidisciplinary collaboration, and addressed health inequalities in line with national policy. While benefits were clear, sustainability, follow-up, and resourcing remain ongoing challenges.

**Disclosure of Interest:**

None Declared

